# FPGA-Based CNN Acceleration on Zynq-7020 for Embedded Ship Recognition in Unmanned Surface Vehicles

**DOI:** 10.3390/s26051626

**Published:** 2026-03-05

**Authors:** Abdelilah Haijoub, Aissam Bekkari, Anas Hatim, Mounir Arioua, Mohamed Nabil Srifi, Antonio Guerrero-Gonzalez

**Affiliations:** 1 Engineering Sciences Laboratory, National School of Applied Sciences of Kenitra, Ibn Tofail University, Kenitra 14000, Morocco; 2 Laboratory of Complex Systems Modeling (LMSC), National School of Applied Sciences of Marrakech, Cadi Ayyad University, Marrakech 40000, Morocco; 3 Laboratory of Research on Sustainable and Innovative Technologies (LaRTID), National School of Applied Sciences of Marrakech, Cadi Ayyad University, Marrakech 40000, Morocco; 4 Department of Automation, Electrical Engineering and Electronic Technology, Universidad Politécnica de Cartagena, 30202 Cartagena, Spain

**Keywords:** FPGA, SWaP, VHDL, CNN, convolutional neural network, Zynq-7020, ship classification, unmanned surface vehicle

## Abstract

Unmanned surface vehicles (USVs) increasingly rely on vision-based perception for safe navigation and maritime surveillance, while onboard computing is constrained by strict size, weight, and power (SWaP) budgets. Although deep convolutional neural networks (CNNs) offer strong recognition performance, their computational and memory requirements pose significant challenges for deployment on low-cost embedded platforms. This paper presents a hardware–software co-design architecture and deployment study for CNN acceleration on a heterogeneous ARM–FPGA system, targeting energy-efficient near-sensor processing for embedded maritime applications. The proposed approach exploits a fully streaming hardware architecture in the FPGA fabric, based on line-buffered convolutions and AXI-Stream dataflow, while the ARM processing system is responsible for lightweight configuration, scheduling, and data movement. The architecture was evaluated using representative CNN models trained on a maritime ship dataset. Our experimental results on a Zynq-7020 system-on-chip demonstrate that the proposed co-design strategy achieves a balanced trade-off between throughput, resource utilisation, and power consumption under tight embedded constraints, highlighting its suitability as a practical building block for onboard perception in USVs.

## 1. Introduction

Unmanned surface vehicles (USVs) are increasingly deployed for missions such as environmental monitoring, bathymetric surveys, and port security, particularly in contexts where continuous human supervision is costly or impractical [1,2]. Recent surveys further highlight the growing integration of heterogeneous sensors and increasing autonomy levels in unmanned marine platforms, reinforcing the need for robust onboard perception to ensure safe and reliable operation [3,4]. Among the available sensing modalities, electro–optical cameras remain attractive because they provide rich semantic information at relatively low cost. However, vision-based ship recognition in real maritime scenes remains challenging due to sea clutter, wakes, reflections, illumination variability, and adverse weather conditions [5].

Deep convolutional neural networks (CNNs) have reshaped visual recognition and detection by enabling powerful hierarchical feature learning that is robust against complex backgrounds and appearance variability [6]. This progress has been consolidated through widely adopted classification backbones and detection frameworks that now serve as practical foundations for many real-world vision systems [7,8,9,10,11,12]. In maritime perception, the availability of public datasets has further advanced this direction by offering realistic ship and open-water imagery captured under diverse conditions [13,14,15]. Building on these benchmarks, recent studies have proposed CNN and YOLO-based approaches tailored to coastal optical scenes, remote-sensing imagery, and UAV viewpoints, demonstrating consistent improvements in maritime situational awareness [16,17,18,19]. An enhanced YOLOv8-based ship detector for USV video streams has also been reported, showing that modern one-stage detectors can provide accurate maritime situational awareness in challenging environments [20].

Despite these advances, deploying CNN inference onboard small and medium-size USVs remains difficult because embedded payloads must respect strict size, weight, and power (SWaP) constraints. High-performance GPUs and dedicated tensor processors provide excellent throughput for vision workloads [21,22,23,24], yet their power consumption, thermal requirements, and system cost do not always match the requirements of compact, long-endurance maritime platforms. This gap motivates energy-aware near-sensor computing solutions, capable of delivering predictable latency and efficient inference on resource-limited hardware [22,25].

Field-programmable gate arrays (FPGAs) offer an attractive alternative for embedded CNN acceleration under tight SWaP constraints. Several accelerators on high-end Xilinx 7-series and UltraScale+ devices combine 8–16-bit fixed-point arithmetic with large DSP budgets to achieve high throughput [26,27,28,29]. Fewer designs target low-cost Zynq-7020 platforms with tighter resources, yet they show that VGG-class networks remain feasible in fixed point [30,31,32]. However, fpgaConvNet [26], Angel-Eye [27], NullHop [28] and mNet2FPGA [30] are mainly reported on ImageNet-like or remote-sensing benchmarks. In contrast, USV-oriented ship-recognition pipelines and fully streaming, hand-written RTL architectures targeting modest SoCs are less frequently addressed in the literature.

To address these constraints, this paper investigated a hardware–software co-design architecture for CNN acceleration on a heterogeneous ARM–FPGA platform, targeting near-sensor processing for embedded ship recognition in unmanned surface vehicles (USVs). The contribution was positioned as a system-level co-design and deployment study on a low-cost Zynq-7020, and it relied on a fully streaming hardware architecture mapped to the FPGA fabric, while the ARM processing system was responsible for lightweight control, configuration, and DMA-based data orchestration. By exploiting line-buffer-based convolutions and AXI-Stream dataflow, the architecture emphasised efficient data re-use and deterministic processing under tight size, weight, and power constraints. Our technical contributions are summarised as follows:

- A fully streaming hardware architecture for CNN inference based on line-buffered convolutions, max-pooling, and fully connected layers, designed for low-latency and memory-efficient processing on resource-constrained FPGA platforms.
- A hardware–software co-design strategy for heterogeneous ARM–FPGA systems, clearly partitioning computation in the programmable logic and control and data movement in the processing system through AXI-Stream and DMA interfaces.
- A comprehensive design-space evaluation on a low-cost Zynq-7020 system-on-chip, including post place-and-route resource utilisation, timing, and power analysis under strict DSP and BRAM constraints.
- An experimental validation using representative CNN models (VGG16, AlexNet, and ResNet-18) for maritime ship recognition, together with a comparison against representative CNN–FPGA accelerators.

## 2. Related Work

Research related to this work can be organised along three complementary directions: unmanned surface vehicles (USVs) and maritime perception systems, ship-detection methods and the public datasets used for evaluation, and FPGA-based acceleration architectures for convolutional neural networks (CNNs). In the following, we review these directions and progressively narrow the focus toward the gap addressed by our Zynq-7020 streaming accelerator for USV-oriented ship recognition.

Early USV developments concentrated on guidance, navigation and control, often assuming simple perception pipelines or human supervision [1,2]. More recent surveys have shown that modern unmanned marine vehicles integrate heterogeneous sensor suites, combining GPS, IMU, radar, LiDAR and electro–optical cameras to support autonomous environmental monitoring, inspection and bathymetric surveys [3,4]. In these platforms, cameras provide rich visual information at relatively low cost, yet they require robust vision algorithms able to cope with sea clutter, wakes, glare, fog and variable illumination, as highlighted in survey work on electro–optical video processing for maritime object detection [5]. Consequently, several recent systems embed deep-learning based detectors in the USV perception stack, typically running on GPU-class boards or high-end embedded SoCs, which motivates the search for more energy-efficient near-sensor computing solutions.

The progress of ship-detection algorithms has been strongly driven by the availability of public datasets. SeaShips offers more than 30,000 images captured by coastal video-surveillance cameras with bounding-box annotations for six common ship categories [13]. MODS targets maritime objects in optical remote-sensing imagery [33], while SeaDronesSee focuses on person and vessel detection in UAV imagery above open water [14]. The Aboships dataset further extends the coverage to near-shore and offshore scenes with diverse viewing angles and challenging lighting conditions [15]. On these datasets, modern object detectors derived from YOLO, SSD and Faster R-CNN have become the dominant approach [9,10,11]. Examples include R-CNN-based ship recognition in high-resolution satellite images [16], YOLOv7 variants tailored to maritime UAV footage [17], and improved YOLO-based pipelines for small-ship detection in optical remote-sensing images [18,19]. In our own previous work, we proposed a fast YOLOv7-based CNN for real-time sea-ship recognition in streaming video [34] and an enhanced YOLOv8 ship detector to support USV collision-avoidance and maritime surveillance tasks [20]. These systems demonstrate that state-of-the-art CNN detectors can achieve accurate ship localisation and classification in operational conditions, although they generally rely on GPUs or powerful embedded processors.

This reliance on powerful compute platforms brings the hardware dimension to the foreground. From a broader perspective, several surveys have reviewed accelerators for deep neural networks and CNNs, emphasising the impact of memory hierarchy, dataflows and quantisation on performance and energy efficiency [21,22,35]. Dedicated tensor processors and GPU architectures deliver very high throughput in datacenters [24], while embedded GPU modules, such as NVIDIA Jetson, offer a compromise between performance and power consumption [23,36]. For FPGAs, comprehensive reviews have discussed the design space of CNN accelerators, including mapping strategies, toolflows and performance trade-offs [37,38,39,40,41,42,43,44,45,46]. Additional work has surveyed FPGA-based accelerators for deep learning with a focus on embedded and edge platforms [25]. A common conclusion has been that FPGAs can deliver high energy efficiency and deterministic latency for CNN inference, provided that arithmetic precision, parallelism and memory access patterns are carefully optimised [21,25,37,39,47].

In line with this conclusion, a large body of work has investigated quantisation and binarisation techniques that are especially attractive for FPGA implementations. Standard 8-bit integer quantisation reduces memory footprints and enables efficient integer-only inference [48]. More aggressive schemes such as XNOR-Net replace multiplications by bitwise operations [49]. Frameworks like FINN and related architectures further implement binarised or low-precision CNNs as deeply pipelined dataflows that exploit the fine-grain reconfigurability of the fabric [50,51].

Beyond quantisation, many authors have focused on micro-architectures and dataflows tailored to convolutional workloads. Eyeriss and Eyeriss v2 investigate weight-stationary and output-stationary schemes supported by sophisticated on-chip memory hierarchies [52,53]. Other works have proposed multi-row line-buffer, loop-tiling and stream-based strategies to minimise off-chip traffic and balance DSP and BRAM usage [54,55,56,57,58]. The fpgaConvNet framework targets both regular and irregular CNN topologies and automatically maps them to FPGA accelerators using analytical performance and resource models [26].

In parallel, several application-oriented accelerators have been demonstrated on Xilinx Zynq and UltraScale+ platforms. ZynqNet shows a hand-optimised ImageNet-scale CNN accelerator on Zynq-7020 [59]. CloudSatNet and CloudSatNet-1 integrate CNN engines into satellite payloads for on-board cloud-cover classification and remote-sensing image analysis [60,61]. Other studies have analysed general FPGA accelerators for deep neural networks [38,39] and have proposed power-aware CNN engines or reconfigurable building blocks tuned to embedded constraints [62]. Additional work has compared streaming and systolic-array dataflows, high-level synthesis and hand-written RTL, and different quantisation strategies for mapping CNNs to Xilinx 7-series and UltraScale+ devices [63]. Together, these contributions show that FPGAs can sustain complex vision workloads within modest power budgets across a wide range of application domains.

Several works are particularly relevant to the present accelerator because they target mid-range Zynq devices. Angel-Eye on Zynq-7045 employs 8-bit fixed-point arithmetic and a highly parallel processing array for VGG16-like workloads [27]. NullHop on Kintex-7 exploits sparsity in CNN feature maps to skip computations and reduce memory transfers [28]. The mNet2FPGA flow maps a fixed-point CNN to a Zynq-7020 SoC using 16-bit arithmetic and a combination of convolution and fully connected engines [30]. Additional architectures on Zynq and UltraScale+ explore heterogeneous CPU–FPGA partitions and energy-efficient inference of large CNNs [29,31,32]. While these designs achieve high GOP/s figures, many of them assume 8–16-bit quantisation, large DSP budgets or high-end devices, and they are often evaluated on generic datasets such as ImageNet rather than on maritime ship-recognition workloads.

In the maritime domain, most recent ship-detection, collision-avoidance and surveillance systems deploy CNN or YOLO-based detectors on GPU-class platforms (desktop GPUs, cloud backends or embedded modules such as Jetson), owing to their mature software ecosystem and high throughput [16,17,18,19]. This is also the case for our previous YOLOv7 and YOLOv8-based ship-detection frameworks for USV video streams, which achieve real-time performance at the cost of relatively high power consumption [20,34]. In contrast, the present work investigated a VHDL-based, fully streaming CNN accelerator on a low-cost Zynq-7020 (ZedBoard), with a 32-bit fixed-point datapath and a line-buffer convolution core, evaluated on VGG16, AlexNet and ResNet-18 trained on SeaShips-derived ship crops. The proposed design is explicitly positioned as a near-sensor processing module for USV ship recognition under tight size, weight and power constraints, bridging the gap between deep maritime perception algorithms and energy-aware embedded implementation.

## 3. System Architecture and Implementation Overview

### 3.1. USV Vision Architecture

The proposed system is designed around a practical USV deployment scenario in which vision-based ship recognition supports navigation and maritime awareness, while respecting the computational and energy constraints of embedded maritime platforms. To keep the perception payload lightweight and energy efficient, the architecture separates mission-critical control from compute-intensive vision processing.

**Figure 1:**
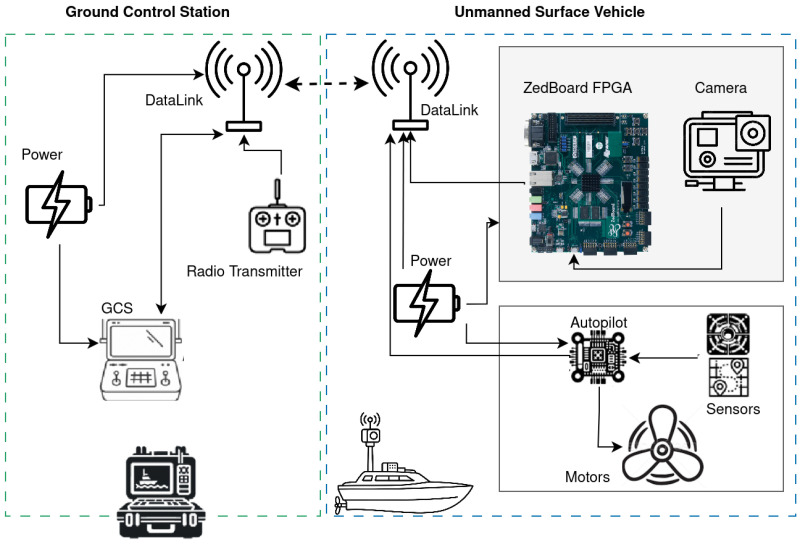
Hardware configuration of the USV and GCS.

As illustrated in Figure 1, the proposed USV platform is organised around a clear separation between the mission subsystem and the payload subsystem. The mission subsystem includes the autopilot and the ground control station (GCS), where the pilot selects the operating mode (manual, assisted, or fully autonomous), monitors telemetry, and issues high-level navigation commands. Guidance, navigation and control of the vessel are therefore handled at the mission level, independently of the vision algorithm.

The payload subsystem is composed of an RGB camera mounted on the USV and a ZedBoard FPGA that implements the CNN-based ship-recognition accelerator. Thanks to the low power consumption of the Zynq-7020 device, this payload can remain continuously active during the mission without significantly affecting the energy budget of the platform. While the USV executes the selected mission mode, each incoming frame is processed on board to recognise ships in the camera field of view. When a vessel is detected, the payload sends an alert message and the associated detection metadata through the datalink to the GCS. The pilot is thus warned to pay attention to the surrounding traffic and can decide how to react (e.g., adjust the route, slow down or switch to manual control). In this configuration, the detection module acts as an always-on safety layer that augments the situational awareness of the human operator without interfering with the primary mission controller.

### 3.2. Implementation Platform and Toolflow

Field-programmable gate arrays (FPGAs) are reconfigurable digital integrated circuits composed of an array of configurable logic blocks (CLBs) surrounded by input/output (I/O) blocks and interconnected by a programmable routing network. CLBs implement combinational and sequential logic, while switch matrices configure the routing so that the fabric behaves as a dedicated hardware circuit once it has been programmed. This fine-grain reconfigurability allows designers to tailor the architecture to a specific application and to exploit massive spatial parallelism, which is particularly attractive for compute-intensive workloads such as the convolutions of a CNN.

Beyond the classical view of FPGAs as reconfigurable logic fabrics (CLBs and routing), modern devices integrate several hardened resources that are key enablers for embedded AI acceleration. First, on-chip memories such as Block RAM (BRAM) (and UltraRAM in newer families) provide predictable, multi-ported buffering for streaming dataflows, enabling line buffers, FIFOs and local weight caches without relying on external DDR for every access. Second, dedicated DSP slices (e.g., DSP48 blocks) offer high-throughput multiply–accumulate operations with optional pre-adders and pipeline registers, which is important for CNN convolutions and fully connected layers. Third, clock-management tiles (PLLs/MMCMs) allow precise frequency generation and stable pipelined designs, while hardened I/O resources support high-bandwidth interfaces and deterministic data movement.

In addition, FPGA–SoC families (e.g., Xilinx Zynq and MPSoC generations) couple programmable logic (PL) with embedded processing systems (PSs), typically ARM cores capable of running full operating systems (e.g., embedded Linux) and managing networking, storage and peripherals. Higher-end SoCs may also integrate real-time processor subsystems (e.g., Cortex-R class cores in MPSoC devices) for time-critical control tasks, and some FPGA families provide multi-gigabit serial transceivers (e.g., GTX/GTH) and hardened interface IP (PCIe, Ethernet, high-speed memory controllers) to enable low-latency sensor/compute interconnects. Finally, vendor IP ecosystems include domain-specific accelerators for deep learning, such as DPU-style neural-network engines and optimised compute libraries, which can be combined with custom RTL/HLS modules depending on the required trade-off between flexibility, timing closure and resource efficiency. In the present work, we targeted a low-cost Zynq-7020 FPGA-SoC; therefore, the implementation specifically leveraged BRAM-based buffering (line buffers and streaming FIFOs), DSP48-based arithmetic where appropriate, and a PS running Linux/PYNQ for configuration and system integration. Features such as real-time cores, multi-gigabit GTX transceivers and DPU-style IP blocks are typically associated with higher-end MPSoC/FPGA families and were not used in our Zynq-7020 prototype.

In this work, the proposed CNN acceleration architecture was deployed on a Xilinx Zynq-7020 system-on-chip mounted on the ZedBoard evaluation platform. The device combines a dual-core ARM processing system (PS) with FPGA programmable logic (PL) interconnected through high-bandwidth AXI interfaces.

Within this heterogeneous architecture, the ARM processing system is responsible for lightweight control, configuration, and communication tasks, while the programmable logic hosts the streaming hardware datapaths that implement the compute-intensive convolution, pooling, and fully connected operations. The ZedBoard provides external DDR memory, Ethernet and camera-compatible I/O, which makes it a representative low-cost platform for prototyping FPGA-based CNN accelerators in maritime environments.

The core of the proposed accelerator, including the line-buffer organisation, the convolution engine and the associated control finite state machines, was implemented as a custom streaming hardware architecture mapped to the FPGA fabric. To ensure precise control over timing, dataflow and resource utilisation under the tight constraints of a low-cost Zynq-7020 platform, the architecture was realised at register-transfer level using VHDL. While alternative design approaches such as Verilog/SystemVerilog or high-level synthesis (HLS) tools can be employed to generate RTL from C/C++ descriptions, recent studies have shown that, alongside high-level synthesis (HLS) approaches, RTL-based implementations remain a relevant and widely used solution for performance-critical streaming pipelines where fine-grained control over buffering, pipelining and scheduling is required [64].

On top of this hardware architecture, the PYNQ framework provides the software layer required to support the hardware–software co-design. The ARM processing system executes Python 3-based control code to configure the accelerator, manage DMA-based data transfers between external memory and the programmable logic, and handle communication with the USV datalink. This clear separation between a programmable, energy-efficient FPGA fabric for computation and a flexible software layer for control and orchestration is consistent with the always-on payload philosophy of the proposed USV perception system.

### 3.3. CNN Benchmark Models for the FPGA Accelerator

**Figure 2:**
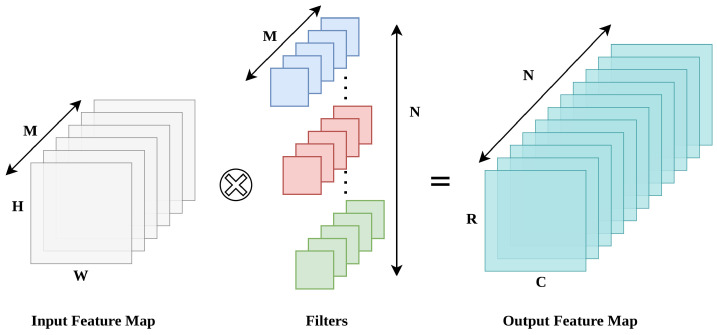
Generic 2-D convolution operation: an input feature map with *M* channels is processed by *N* filters, producing an output feature map of size R×C with *N* channels.

Convolutional neural networks (CNNs) process an input image through a sequence of convolution layers that transform it into increasingly abstract feature maps. At each layer, an input tensor of size H×W with *M* channels is convolved with a bank of *N* learned filters, producing an output tensor of size R×C with *N* channels. This generic operation is depicted in Figure 2. For every spatial position and every output channel, the hardware must perform $K^{2}M$ multiply–accumulate operations, and the total number of MACs grows with the spatial resolution and the number of channels. On a resource-limited FPGA such as the Zynq-7020, where DSP slices, BRAM blocks and external-memory bandwidth are all constrained, it is therefore not realistic to deploy arbitrarily large networks. Instead, it is more practical to evaluate the accelerator with a set of standard CNN models that span different computational footprints and memory requirements.

In this work, we evaluated the convolutional accelerator with three well-known CNN architectures that are widely used in computer vision. Our objective was to benchmark the proposed hardware on standard workloads before integrating it into the USV perception pipeline. Using established models also made it easier to compare our results with previous FPGA-based accelerators.

VGG16 was selected as a deep reference network with a high computational and memory cost. The VGG16 architecture relies on stacks of 3×3 convolutions combined with 2×2 max-pooling layers [8]. Because of its heavy workload, VGG16 remains a common benchmark for FPGA implementations. It stresses both the compute resources and the external-memory bandwidth.

AlexNet was included as a lighter baseline. The AlexNet architecture consists of five convolutional layers followed by three fully connected layers [7]. It was among the first deep CNNs to demonstrate a major breakthrough on ImageNet. Compared with VGG16, AlexNet has a lower computational cost per image [7]. This makes it useful for exploring higher frame rates and more aggressive resource sharing on the FPGA.

ResNet-18 provided a compact and modern reference point. It is widely used as a backbone for image classification and detection [12]. Its residual design offers a good trade-off between accuracy and complexity. For FPGA mapping, ResNet-18 also represents an intermediate workload between the heavier VGG16 and the lighter AlexNet [12].

All three models operate on RGB images. They can be used as standalone classifiers or as feature-extraction backbones for detection heads in YOLO-based pipelines. In this study, our main purpose was to use them as reference workloads. This allowed us to validate the proposed streaming hardware architecture on the Zynq-7020 platform and to demonstrate its suitability as a near-sensor processing building block for future USV perception systems.

### 3.4. Maritime Ship Dataset for USV Perception

Publicly available ship datasets have become essential for training and benchmarking vision algorithms in maritime applications. They provide annotated images of vessels under diverse conditions and support realistic evaluation of models intended for USV perception. Representative examples include the SeaShips dataset [13] and the Aboships near-shore and offshore dataset [15].

In this study, we adopted SeaShips, which we also used in our previous YOLOv8-based USV detection work [20]. SeaShips was collected from a coastal video-surveillance network comprising 156 cameras deployed at 50 locations along the north-western coast of Hengqin Island, covering approximately 53 km^2^. Each location included one low-light HD camera and two HD bullet cameras. From this imagery, we selected ship instances and organised them into six semantic classes: ore carriers, bulk cargo carriers, container ships, general cargo ships, fishing boats and passenger ships [13].

To align the dataset with the benchmark CNN models used in the previous subsection, we converted SeaShips into a classification-style ship-crop dataset. For each annotated vessel, a crop centred on the bounding box was extracted and resized to 224×224×3, matching the input resolution of VGG16, AlexNet and ResNet-18. When multiple ships appeared in the same frame, we generated one crop per instance, each inheriting its corresponding class label. The ship crops were then split into training, validation and test subsets with approximate proportions of 70%, 15% and 15%, while preserving class representation across all splits.

The resulting dataset, which was used to train VGG16, AlexNet and ResNet-18, contained 9223 ship crops, and its class distribution is summarised in Table 1.

The CNN models (VGG16, AlexNet, and ResNet-18) are trained on the SeaShips-derived ship-crop dataset using a standard transfer-learning procedure. Each network is initialized with ImageNet-pre-trained weights, then fine-tuned on the ship-crop training set, while the validation split is used for model selection and the test split for final evaluation.

## 4. Design of the CNN Accelerator

**Figure 3:**
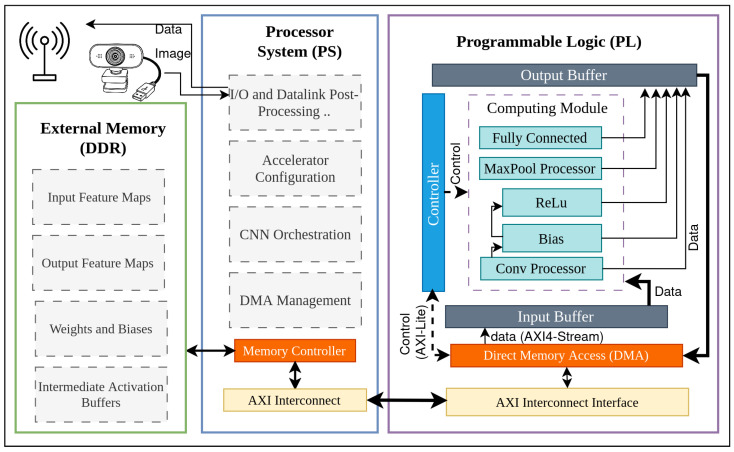
System-level architecture of the proposed CNN accelerator implemented on a ZedBoard (Zynq-7020).

The proposed accelerator is implemented on a Xilinx Zynq-7020 SoC hosted on a ZedBoard evaluation kit. The design follows a streaming hardware/software co-design in which the ARM processing system (PS) is used exclusively for control, orchestration and communication, while the programmable logic (PL) executes all compute-intensive CNN operations. At the highest level, the system is organised around three main domains: external DDR memory, the PS and the PL, as shown in Figure 3.

**Figure 4:**
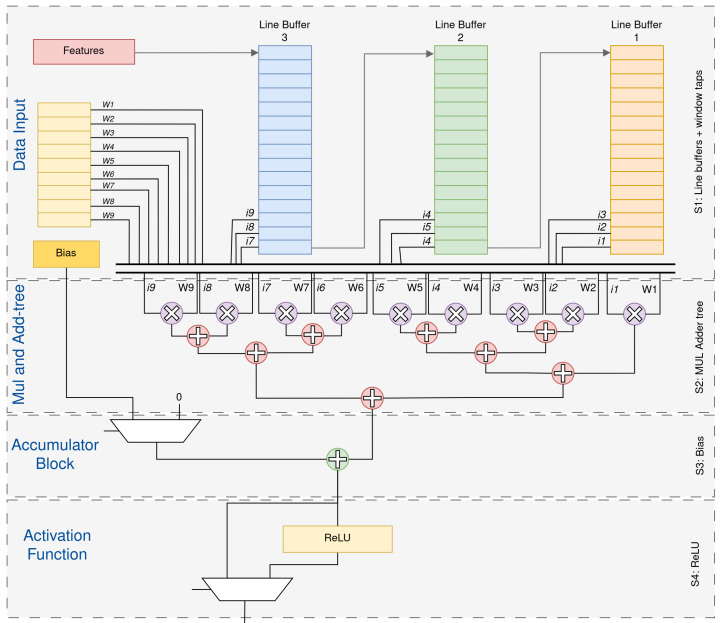
Micro-architecture example of the convolution core for a kernel with nine coefficients (e.g., a 3×3 filter).

External DDR is shared between PS and PL and stores input and output feature maps, convolution and fully connected weights and biases, and intermediate activation buffers. The PS runs a PYNQ-based runtime to configure the accelerator, manage AXI DMA transfers and perform I/O and datalink post-processing for the USV. The PL contains a computing module that performs convolutions, pooling and fully connected operations in a pipelined fashion. Inside this module, the convolution engine relies on line buffers and a multiply–accumulate tree; Figure 4 illustrates a representative instance of this core configured for a kernel with nine coefficients (e.g., a 3×3 filter), which is re-used across network layers by changing the control parameters and weights.

### 4.1. System-Level Architecture

**Figure 5:**
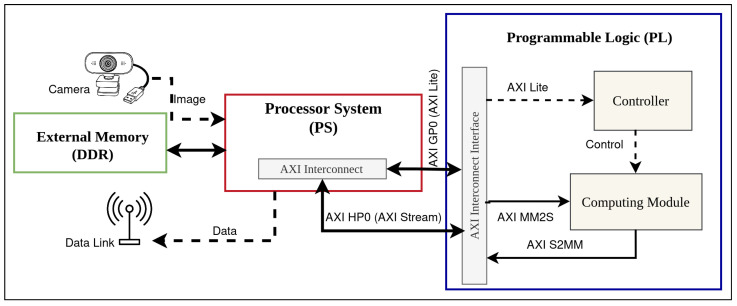
Data flow overview of the Zynq-7020 PS/PL co-design.

Figure 5 provides a control–data flow overview of the proposed PS/PL co-design on Zynq-7020. It highlights the separation between the control path (PS configuration through AXI-Lite toward the PL controller) and the data path (feature maps and parameters transferred between DDR and the PL through AXI HP0 and the DMA MM2S/S2MM channels).

Figure 3 summarises the system-level organisation. External DDR memory stores input and output feature maps, convolution and fully connected weights and biases, and intermediate activation buffers. The DDR controller and AXI interconnect in the Zynq processing system expose this memory to both the PS and the PL.

On the software side, the PS runs a lightweight PYNQ-based runtime. Among its tasks are: (i) accelerator configuration, by writing layer parameters into AXI-Lite control registers in the PL; (ii) CNN inference orchestration, by sequencing convolution, pooling and fully connected phases for each layer of VGG16, AlexNet and ResNet-18; (iii) memory transfer management, by setting up and monitoring AXI DMA transfers between DDR and the PL through the high-performance AXI ports; and (iv) I/O and datalink post-processing, which includes image acquisition, extraction of the final classification scores and transmission of compact results to the USV control system over the communication link.

The PL implements the compute-intensive part of the network as a fully streaming datapath. An AXI DMA core in the PL reads feature maps and weights from DDR and writes back processed feature maps and classification scores. The DMA feeds an input buffer that delivers a continuous stream of pixels to the computing module, and collects results from an output buffer before they are returned to DDR. A dedicated controller block decodes the configuration registers set by the PS and generates the internal control signals for the convolution, pooling and fully connected units, as well as for the DMA engine.

### 4.2. Micro-Architecture of Convolution Core

The internal micro-architecture of the convolution processor is illustrated in Figure 4. The example corresponds to a kernel with nine weights, such as a 3×3 filter, although the same structure is re-used across all convolutional layers by changing the control parameters and the weight set.

Incoming pixels from the current input feature map are first written into an input stream buffer in the PL. This buffer delivers a regular one-sample-per-cycle stream to the convolution core. At the core input, three line buffers implemented in on-chip memory store three consecutive rows of the feature map. At each clock cycle, the taps of these line buffers provide nine spatially adjacent samples $\left\{i_{1},\ldots ,i_{9}\right\}$ that form the current 3×3 convolution window around the pixel being processed.

In parallel, the corresponding kernel weights $\left\{w_{1},\ldots ,w_{9}\right\}$ for the active output channel are fetched from on-chip weight storage. The nine input samples and nine weights are then processed by a parallel multiply–accumulate (MAC) tree: nine multipliers compute the products $i_{k}w_{k}$, and a balanced adder tree reduces these partial products to a single sum. A bias term $b_{o}$ associated with the current output channel *o* is added to this sum.

To support multi-channel convolutions, an accumulator stage is placed after the adder tree. For each spatial position (x,y) and output channel *o*, the accumulator either resets (at the start of the input-channel loop) or accumulates partial sums over all input channels $c=0,\ldots ,C_{\mathrm{in}}-1$, implementing the discrete convolution:

(1) $$y_{o}\left(x,y\right)=b_{o}+\sum_{c=0}^{C_{\mathrm{in}}-1}\sum_{m=0}^{K-1}\sum_{n=0}^{K-1}w_{o,c,m,n},x_{c}\left(x+m-\Delta_{x},\;y+n-\Delta_{y}\right),$$

where K=3 in the present design and $\left(\Delta_{x},\Delta_{y}\right)$ encode the padding offset introduced by the input-stream buffer.

The accumulated value is then passed through a ReLU activation unit, which clips negative results to zero. All arithmetic in this pipeline uses a 32-bit fixed-point format, identical to the rest of the CNN datapath. Depending on the layer configuration, the activated outputs are either forwarded directly to the max-pooling unit within the computing module or written to the output buffer to be sent back to DDR.

Once the line buffers are primed, this micro-architecture produces one output sample per clock cycle for each active output channel, achieving a constant throughput limited only by the clock frequency and the available parallelism in the MAC tree.

### 4.3. Line-Buffer-Based Input Dataflow and Memory Optimisation

The convolution engine is organised as a streaming datapath based on line buffers in order to exploit spatial re-use and minimise external-memory traffic. For a given 2-D convolutional layer, we adopt the following notation. The input feature-map tensor is:

$$X\in R^{C_{\mathrm{in}}\times H\times W},$$

where $C_{\mathrm{in}}$ is the number of input channels and *H* and *W* are the input height and width, respectively; $x_{c}\left(x,y\right)$ denotes the sample of channel *c* at spatial location (x,y). The kernel tensor is

$$W\in R^{C_{\mathrm{out}}\times C_{\mathrm{in}}\times K\times K},$$

where $C_{\mathrm{out}}$ is the number of output channels and *K* is the kernel size (here K=3). Each output channel *o* has a K×K kernel $w_{o,c,m,n}$ for every input channel *c*. The corresponding output feature-map tensor is:

$$Y\in R^{C_{\mathrm{out}}\times H_{\mathrm{out}}\times W_{\mathrm{out}}}$$

where $H_{\mathrm{out}}$ and $W_{\mathrm{out}}$ are the height and width of the output feature maps after padding and stride. For such a layer, the total number of multiply–accumulate operations is:

(2) $$N_{\mathrm{MAC}}=H_{\mathrm{out}},W_{\mathrm{out}},C_{\mathrm{out}},C_{\mathrm{in}},K^{2}$$

If a complete network requires $N_{\mathrm{MAC}}^{\left(\mathrm{net}\right)}$ MACs per image and the measured frame rate is FPS, the effective throughput in MAC/s and GOP/s is:

(3) $${\mathrm{Throughput}}_{\mathrm{MAC}/s}=N_{\mathrm{MAC}}^{\left(\mathrm{net}\right)}\times \mathrm{FPS}\;$$

(4) $${\mathrm{Throughput}}_{\mathrm{GOP}/s}=\frac{2,N_{\mathrm{MAC}}^{\left(\mathrm{net}\right)}\times \mathrm{FPS}}{10^{9}}$$

where one MAC is counted as two elementary operations (one multiplication and one addition).

On the data-movement side, input feature maps and kernel weights are fetched from DDR by the AXI DMA and stored in on-chip buffers in the PL. The input stream buffer plays a central role: it converts burst-oriented AXI transactions into a regular one-sample-per-cycle stream for the convolution core and embeds the control logic for padding and stride. This means that padding and stride are handled locally in hardware, without extra software overhead and without materialising padded feature maps in DDR.

**Figure 6:**
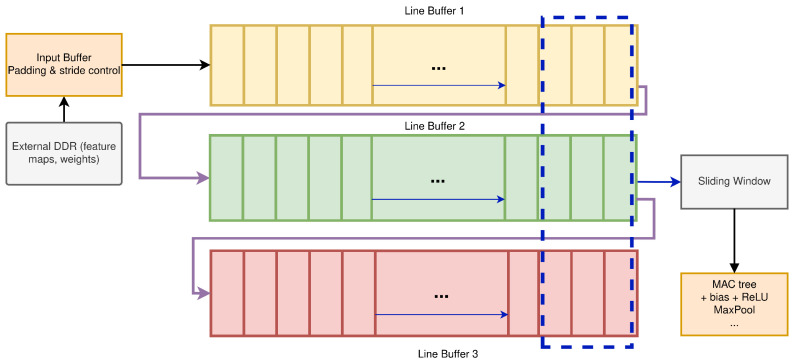
Line-buffer-based streaming dataflow for a 3×3 convolution window.

For a 3×3 kernel, the convolution engine uses three cascaded line buffers of length *W*. At each clock cycle, a new pixel from the input stream is written into the last position of the third (bottom) line buffer. The contents of the line buffers are shifted, so that the sample leaving the third buffer is injected into the last position of the second buffer, and the sample leaving the second buffer is injected into the last position of the first buffer. In this way, the three buffers always hold three vertically aligned rows of the feature map. At each clock cycle, the taps at the current column index provide the 3×3 window $\left\{i_{1},\ldots ,i_{9}\right\}$, as shown in Figure 6. When the window slides horizontally by one column, eight of the nine samples are re-used from the previous position and only one new pixel is fetched from the input stream. As a result, each input pixel is read once from external DDR and then reused locally as the kernel window moves across the feature map.

Padding is implemented by the input-buffer controller, which suppresses reads outside the valid image region and injects zeros on the corresponding line-buffer taps; no padded pixels are stored in external memory. Stride s>1 is realised by skipping window evaluations or advancing the stream and line-buffer pointers so that only one out of *s* positions produces a valid output. Both mechanisms rely solely on local address-generation and enable logic and do not require additional passes through DDR or extra PS intervention.

Compared with a naïve sliding-window implementation without line buffers, which would require $K^{2}$ input reads from DDR for every output sample, the proposed organisation reduces input-feature-map reads to one new pixel per horizontal step of the window. For the 3×3 kernels used in this work (K=3), this corresponds to an ideal reduction of external reads by approximately a factor of nine. When combined with the fact that padding is not materialised in DDR and that strided positions are discarded on the fly, the overall DDR traffic per layer is close to the theoretical minimum (one read per input pixel and one write per output pixel). This reduction in memory bandwidth directly lowers latency and dynamic energy and allows the convolution engine to sustain a constant one-sample-per-cycle throughput within the resource and power constraints of the ZedBoard (Zynq-7020). At the compute level, in contrast to iterative convolution engines that re-use a single multiply–accumulate (MAC) unit to process all kernel coefficients sequentially, the proposed convolution core implements the 3×3 kernel fully in parallel. Once the line buffers are primed, it produces one output sample per clock cycle and per active output channel, whereas a sequential MAC-based design would require roughly $K^{2}$ cycles per output for a K×K kernel (nine cycles in the 3×3 case). The presented architecture therefore trades a higher number of multipliers for a significantly lower number of cycles per output pixel, favouring throughput over minimal resource usage, which is well aligned with the real-time constraints of embedded vision on a resource-limited Zynq-7020 device, and this trade-off between resource usage and throughput is summarised in Table 2.

### 4.4. Fixed-Point Parameters and DDR Memory Mapping

To improve reproducibility and portability, this subsection details how trained parameters (weights and biases) are exported from the training framework and deployed into the Zynq-7020 external DDR memory, then streamed to the programmable logic (PL) through AXI DMA. In our hardware–software co-design, the processing system (PS) running PYNQ performs: (i) fixed-point conversion, (ii) deterministic tensor serialisation into linear DDR buffers, and (iii) generation of layer-wise transfer descriptors and control words, while the PL consumes feature maps and parameters as a streaming dataflow.

#### 4.4.1. Fixed-Point Format

All CNN parameters and activations are encoded in a signed 32-bit fixed-point datapath using the Q15.16 format. This precision was selected to ensure safe numerical range during accumulation-heavy layers on a resource-limited Zynq-7020, where intermediate sums can exceed the dynamic range of narrower formats unless additional scaling is introduced. Using Q15.16 allows us to keep a uniform scaling factor across the full PS/PL streaming pipeline, with explicit rounding and saturation rules, which makes the hardware behaviour deterministic and the deployment reproducible (consistent tensor quantisation, DDR packing, and AXI–DMA transfers) in the current prototype. A real value x∈R is mapped to an integer $x_{\mathrm{fx}}\in Z$ as:

(5) $$x_{\mathrm{fx}}=\mathrm{sat}\left(\left\lfloor x\cdot 2^{16}\right\rceil \right),$$

where ⌊·⌉ denotes rounding to nearest and sat(·) saturates to the signed 32-bit range. During inference, multiplying two Q15.16 values produces a Q30.32 product. In the proposed datapath, partial products are accumulated with sufficient guard bits, and the result is rescaled back to Q15.16 by a right shift of 16 bits (with rounding), while saturation is applied at stage outputs to prevent overflow. This ensures deterministic fixed-point arithmetic consistent with the 32-bit datapath reported in Section 5.

#### 4.4.2. Weight Tensor Layout and Packing Order

For a convolutional layer with $C_{\mathrm{in}}$ input channels, $C_{\mathrm{out}}$ output channels and a $K_{y}\times K_{x}$ kernel, weights are stored in DDR as a flattened 1-D array following the explicit indexing order:

(6) $$W\left[o,i,k_{y},k_{x}\right],\;o\in \left[0,C_{\mathrm{out}}-1\right],\;i\in \left[0,C_{\mathrm{in}}-1\right],\;k_{y}\in \left[0,K_{y}-1\right],\;k_{x}\in \left[0,K_{x}-1\right].$$

This order is aligned with the PL nested loops that accumulate partial sums over the input-channel dimension while re-using the $K_{y}\times K_{x}$ window produced by the line buffers (Section 4). With this layout, all coefficients required for a given output channel *o* are contiguous in memory, which enables burst-friendly DMA transfers.

The corresponding 1-D address (in number of 32-bit words) is:

(7) $$\mathrm{idx}\left(o,i,k_{y},k_{x}\right)=\left(\left(\left(o\cdot C_{\mathrm{in}}+i\right)\cdot K_{y}+k_{y}\right)\cdot K_{x}+k_{x}\right).$$

Biases are stored as a contiguous vector b[o] in Q15.16, one 32-bit word per output channel.

#### 4.4.3. DDR Memory Regions

We allocate contiguous DDR regions for images/feature maps, layer parameters, and intermediate results. In our case, PYNQ allocates physically contiguous buffers, and the PS passes their base physical addresses to the AXI DMA engine and the PL control registers. Table 3 summarises the memory map used by the runtime.

#### 4.4.4. Runtime Interface from Model Files to Board Memory

Trained parameters are exported offline and loaded by the PS. The PYNQ runtime performs: (i) float-to-Q15.16 conversion, (ii) flattening with the order in (6), and (iii) DMA descriptor preparation. A simplified pseudocode is provided below:

# PYNQ-side pseudocode (simplified)

# Load trained parameters (float)

W_float, b_float = load_layer_params(layer_id)     # W:[Cout,Cin,Ky,Kx],

b: [Cout]

# Quantize to 32-bit fixed-point Q15.16

W_fx = float_to_q1516(W_float)                     # int32 (Eq. q1516)

b_fx = float_to_q1516(b_float)                     # int32

# Allocate CMA-contiguous DDR buffers

W_buf = allocate(shape=(W_fx.size,), dtype=int32)  # 1-D contiguous

b_buf = allocate(shape=(b_fx.size,), dtype=int32)

# Serialize weights in (o,i,ky,kx) order (matches Eq. w_flat)

W_buf[:] = W_fx.ravel(order=’C’)                   # [o,i,ky,kx]

b_buf[:] = b_fx

# Cache maintenance before DMA

W_buf.flush(); b_buf.flush()

fm_in_buf.flush()

# Program PL control registers (layer config + base addresses)

write_ctrl_regs(layer_cfg, W_base=W_buf.physical_address,

B_base=b_buf.physical_address)

# Stream feature maps via AXI DMA (HP0)

dma.sendchannel.transfer(fm_in_buf)                # MM2S

dma.recvchannel.transfer(fm_out_buf)               # S2MM

dma.sendchannel.wait(); dma.recvchannel.wait()

This explicit description complements Section 4 and provides the missing link between trained model files and the physical memory mapping used by the Zynq-7020 DMA-based deployment.

### 4.5. DMA Data Movement and Bandwidth Analysis

The proposed ARM–FPGA co-design streams feature maps and CNN parameters between external DDR and the programmable logic (PL) through an AXI DMA engine connected to the PS HP0 high-performance port (64-bit), as described in Section 5. Since the accelerator is designed as a fully streaming datapath, data movement is a first-order factor for end-to-end latency. Accordingly, we detail the data exchanged with DDR during each layer execution, derive the corresponding traffic in bytes, and we relate these transfers to the compute and orchestration contributions observed in the end-to-end latency breakdown.

#### 4.5.1. Per-Layer DDR Traffic Model

We consider a 2-D convolution layer with input tensor $X\in R^{C_{\mathrm{in}}\times H\times W}$, output tensor $Y\in R^{C_{\mathrm{out}}\times H_{\mathrm{out}}\times W_{\mathrm{out}}}$, and kernel tensor $W\in R^{C_{\mathrm{out}}\times C_{\mathrm{in}}\times K_{y}\times K_{x}}$. In our implementation, all activations, weights and biases are stored in DDR as 32-bit fixed-point words (Q15.16); hence, S=4 bytes/value. Under the layer-wise execution used in this work (configure a layer, stream its inputs/parameters, write back its outputs), the dominant DDR traffic of a convolution layer is approximated by:

(8) $$T_{\mathrm{DDR}}^{\left(\mathrm{conv}\right)}\approx S\cdot \left(\underset{\mathrm{read}\;\mathrm{input}\;\mathrm{feature}\;\mathrm{map}}{\underset{︸}{C_{\mathrm{in}}HW}}+\underset{\mathrm{write}\;\mathrm{output}\;\mathrm{feature}\;\mathrm{map}}{\underset{︸}{C_{\mathrm{out}}H_{\mathrm{out}}W_{\mathrm{out}}}}+\underset{\mathrm{read}\;\mathrm{weights}}{\underset{︸}{C_{\mathrm{out}}C_{\mathrm{in}}K_{y}K_{x}}}+\underset{\mathrm{read}\;\mathrm{biases}}{\underset{︸}{C_{\mathrm{out}}}}\right).$$

This model reflects the actual run-time behaviour of the PYNQ-controlled prototype: input feature maps and parameters are streamed from DDR to the PL (MM2S), and the resulting feature maps are streamed back to DDR (S2MM). The “≈” sign indicates that DMA descriptor overheads and burst padding/alignment effects are not counted.

For max-pooling, no parameters are transferred and the layer performs a single read and write of feature maps. Since channel count is preserved by pooling ($C_{\mathrm{out}}=C_{\mathrm{in}}=C$), the DDR traffic is:

(9) $$T_{\mathrm{DDR}}^{\left(\mathrm{pool}\right)}\approx S\cdot \left(\underset{\mathrm{read}\;\mathrm{input}\;\mathrm{feature}\;\mathrm{map}}{\underset{︸}{C,HW}}+\underset{\mathrm{write}\;\mathrm{pooled}\;\mathrm{feature}\;\mathrm{map}}{\underset{︸}{C,H_{\mathrm{out}}W_{\mathrm{out}}}}\right).$$

#### 4.5.2. On-Chip Buffering and AXI DMA Transfer

The convolution core is a streaming architecture based on three line buffers of length *W* that generate a 3×3 sliding window (Figure 6). With 32-bit Q15.16 words, one line buffer requires W·S bytes and the three-line storage is 3WS bytes, re-used as the window slides. Padding and stride are handled locally by the PL controller, so padded feature maps are not materialised in DDR and no extra DDR traffic is introduced for border handling.

On the PS side, PYNQ allocates DMA buffers as physically contiguous DDR regions, and explicit cache maintenance (flush/invalidate) is performed to ensure coherent DMA transactions. Because HP0 carries 64-bit beats while tensors are stored as 32-bit words, each beat transports two Q15.16 values when buffers are contiguous and aligned, which supports efficient burst transfers and high bus utilisation.

#### 4.5.3. Streaming Overlap and Latency Breakdown

Within a layer execution, the proposed streaming datapath enables a natural overlap between computation and data movement: after line-buffer priming, the PL starts producing valid outputs and sustains a steady one-sample-per-cycle stream while the AXI DMA continues feeding the remaining input and parameter words from DDR. In the current prototype, however, layer execution is orchestrated sequentially by the PS for each stage (configure → stream → write-back) for Conv → Pool and FC phases, and cross-layer pre-fetching/double-buffering is not yet implemented. Consequently, the end-to-end latency includes both PL compute time and a non-negligible contribution from DDR transfers and PS-side orchestration.

This behaviour is made explicit by the latency breakdown of Table 6. In particular, DMA data transfers correspond to the payload streaming time on the HP0 interface (MM2S and S2MM) for feature maps and parameters, whereas “PS control” aggregates DMA descriptor setup, AXI-Lite register programming, and Python-level orchestration under PYNQ (including the cache flush/invalidate operations required for coherent DMA). This separation supports the near-sensor argument: the dominant fraction of the execution time remains in PL computation (Conv/Pool + FC), while the remaining overhead is attributable to data movement and orchestration, which can be reduced through improved transfer scheduling and layer-to-layer overlap (e.g., double buffering and pre-fetching).

## 5. Results

The proposed CNN accelerator was implemented in VHDL on the Zynq-7020 SoC. All compute-intensive kernels (convolution, bias addition, ReLU, max-pooling, and the fully connected (FC) block) were described in VHDL and mapped to the programmable logic (PL). Synthesis, place-and-route and bitstream generation were carried out with Xilinx Vivado 2018.3. On the processing system (PS) side, we used the PYNQ Linux distribution adapted to the ZedBoard platform, and the accelerator was controlled by Python scripts running on this PYNQ image. The PL operated at 100 MHz, and all arithmetic in the CNN datapath used a 32-bit fixed-point format. Communication between the PS and PL relied on an AXI-Lite register interface for configuration and on the HP0 AXI high-performance port (64-bit data width) connected to an AXI DMA engine for streaming feature maps from and to external DDR memory.

**Figure 7:**
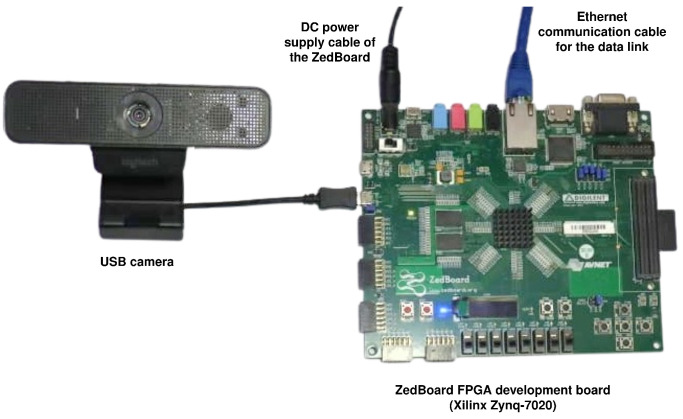
Experimental setup of the proposed accelerator on the ZedBoard (Zynq-7020).

Figure 7 shows the experimental setup corresponding to the hardware architecture discussed above, including the ZedBoard, the USB camera used as an image source, and the Ethernet link for data and control.

### 5.1. Implementation and Resource Utilisation

The architecture of the PL comprises a convolution engine based on line buffers feeding a multiplication-and-add tree, followed by a bias addition block and a ReLU activation unit, a max-pooling unit, and a fully connected block implementing parallel MAC operations per cycle. Around these compute cores, the design also includes input line buffers for feature-map buffering, an AXI-Stream master emulator for test and verification, an AXI DMA interface on the HP0 port for data transfers to and from external DDR, and an AXI-Lite control block that exposes configuration registers to the PS. All compute blocks are interconnected by AXI-Stream interfaces, while the PS communicates with the accelerator through AXI-Lite (for configuration) and AXI DMA on HP0 (for moving input and output feature maps).

Post place-and-route resource utilisation is summarised in Table 4. The design occupies 45,035 LUTs (84.65% of the available LUTs), 44,306 flip-flops (41.64%), 2523 LUTRAM elements (14.50%), and 58 BRAM36 blocks, corresponding to approximately 2.04 Mbit of on-chip memory (41.43% of the BRAM resources). Only 36 DSP48E1 slices (16.36%) are required to implement the convolution, pooling and FC units. The timing report confirms that the design meets the 100 MHz constraint with positive slack, and the post-implementation power estimate from Vivado is approximately 2.04 W. Overall, the accelerator fits within the modest resources and power envelope of the Zynq-7020, leaving a margin of DSPs and BRAMs for additional pre- and post-processing modules if required by the application.

Although the design meets timing at 100 MHz, the current implementation was primarily LUT/routing-limited (84.65% LUT utilisation), rather than constrained by DSP/BRAM availability, which reduced the placement margin for adding further parallel compute engines on the same Zynq-7020 fabric. The selected 100 MHz clock was a conservative timing-closure choice to ensure stable end-to-end PS/PL operation (AXI DMA streaming and AXI-Lite control under PYNQ), rather than a claimed maximum frequency. Consequently, scaling on the same device focused on reducing LUT pressure and routing complexity (e.g., DSP-oriented restructuring of MAC operations, localised pipelining and floorplanning) and on improving PS–PL intercommunication/overlap; higher degrees of parallelism can be achieved by porting the same streaming datapath to a device with a larger LUT budget.

### 5.2. End-to-End Latency and Frame Rate

The performance evaluation targeted three representative CNN models at an input resolution of 224 × 224 × 3: VGG16, AlexNet and ResNet-18. The PL followed a streaming dataflow: for each spatial resolution in the network (224 × 224, 112 × 112, 56 × 56, 28 × 28, 14 × 14), the PS configured the convolution core via AXI-Lite, it set up the AXI DMA to stream input feature maps through HP0, and it collected the convolved feature maps back in DDR before launching the corresponding max-pooling stage. For VGG16, this procedure was repeated for all 13 convolutional layers, each mapped to a single Conv → Pool block, followed by three fully connected layers implemented in PL.

A cycle-accurate analytical model of the streaming architecture was developed and validated both against VHDL testbenches and against measurements on the PYNQ platform. At testbench level, AXI-Stream beats and TLAST events were counted in the first layers to derive the number of clock cycles required for streaming, priming and flushing. On the real Zynq-7020 board, timestamps were collected on the PS side before and after each Conv → Pool and FC phase using PYNQ instrumentation, and the measured times matched the analytical predictions within a small margin.

Based on this combined testbench-and-hardware methodology and assuming an effective computational throughput of 4.45 GMAC/s (8.9 GOPS, with one MAC counting as two operations), the end-to-end latency for VGG16, from an input frame stored in DDR to the final classification scores written back to DDR, was approximately 3.48 s, corresponding to an end-to-end throughput of 0.29 frames per second (FPS).

The same methodology was applied to AlexNet and ResNet-18. In our PYNQ-based prototype, the PS-side orchestration path (AXI-Lite configuration, DMA setup, and cache maintenance) remained unchanged across the networks; therefore, the PS/DMA contribution was treated as a first-order runtime overhead dominated by the control flow, while the PL compute time was scaled according to the theoretical MAC cost (GMAC) of each model. Table 5 reports the GMAC per image, end-to-end latency and resulting FPS for the three networks. For AlexNet, whose complexity is approximately 0.724 GMAC per image at 224×224, the end-to-end latency was 163 ms, corresponding to a throughput of about 6.15 FPS. For ResNet-18, with a cost of roughly 0.90 GMAC per image, the latency was 202 ms and the resulting throughput was around 4.94 FPS.

**Figure 8:**
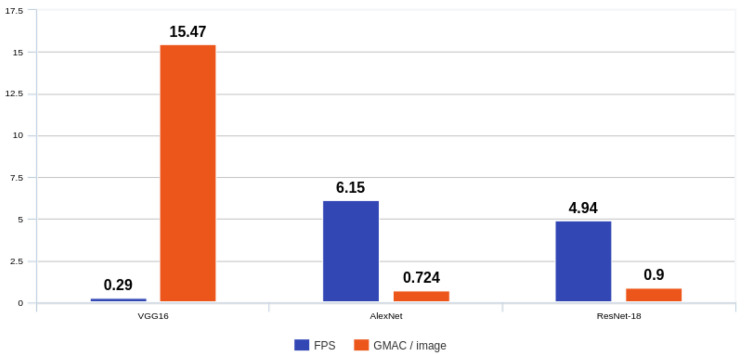
End-to-end FPS and GMAC/image for VGG16, AlexNet and ResNet-18.

Our comparison across the models is summarised in Figure 8, which reports for each network the end-to-end frame rate (FPS) and the corresponding effective throughput in GMAC/s. As expected, the lighter networks converted the same compute budget into higher FPS: AlexNet reached about 6.15 FPS and ResNet-18 about 4.94 FPS, whereas VGG16 ran at approximately 0.29 FPS due to its much larger computational cost per image. In all three cases, the effective throughput was close to 4.45 GMAC/s (8.9 GOPS).

GMAC denoted $10^{9}$ multiply–accumulate (MAC) operations, where one MAC corresponded to one multiplication followed by one addition.

### 5.3. Latency Breakdown for VGG16

To better understand the performance bottlenecks of the presented architecture, the end-to-end inference latency of VGG16 was decomposed into five main components, as reported in Table 6. The time attributed to the programmable logic (PL) included the execution of convolution and pooling operations across all 13 Conv → Pool blocks, as well as the three fully connected layers. The DMA data-transfer term corresponded to the pure streaming time over the AXI HP0 interface (MM2S/S2MM) using 64-bit data beats. Processing-system (PS) control accounted for DMA configuration, AXI-Lite register accesses, and Python-level function calls. Finally, the image I/O term aggregated the input image loading from the external DDR memory and result write-back operations.

The breakdown indicates that convolution and pooling operations executed in the PL account for approximately 1534 ms (44.1%) of the total inference latency, while the fully connected layers contributed about 972 ms (28.0%). Data movement through the AXI HP0 interface required 488 ms (14.0%), PS-side control operations represented 374 ms (10.8%), and image I/O contributed 108 ms (3.1%). Overall, around 72% of the total latency was spent on PL-based computation, whereas the remaining 28% was associated with memory transfers and software-related overheads.

**Figure 9:**
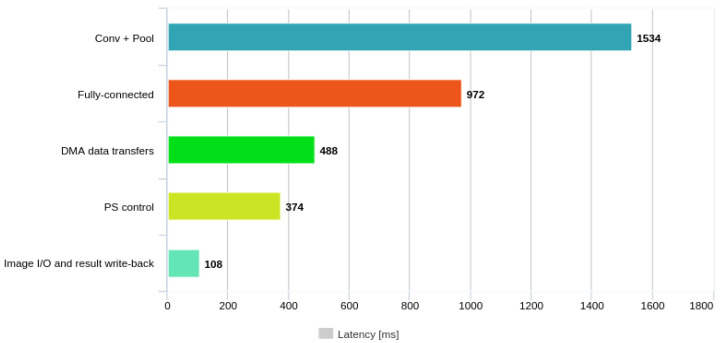
Latency breakdown for a VGG16 inference on the Zynq-7020.

This distribution is visualised in Figure 9, which presents a bar chart of the VGG16 latency components. The figure highlights that the convolution and pooling engine dominated the overall inference latency, while the interaction between the processing system and AXI DMA transferred still accounted for a non-negligible fraction of the total execution time.

For VGG16, the overall inference latency was therefore primarily dominated by convolution and pooling computations due to the depth of the network and the large number of feature maps. The fully connected layers contributed a smaller but noticeable portion of the execution time, whereas the AXI DMA transfers and processing-system control introduced additional overheads that remained secondary compared to the computational workload mapped to the FPGA fabric.

### 5.4. Power Consumption and Energy Efficiency

For USV onboard deployment, energy efficiency was a key metric in addition to raw throughput, since the perception payload had to operate under strict energy and endurance constraints. We therefore complemented the performance results with a power and efficiency analysis. In this work, we report the power estimate of the proposed accelerator, which is 2.04 W at 100 MHz (Table 4). Using the measured effective throughput of 8.9 GOP/s, the resulting energy efficiency was approximately 4.36 GOP/s/W. To facilitate comparison with the literature, Table 7 includes power and energy-efficiency figures whenever they are available in the referenced works, as reported by the original authors.

Finally, in the Section 6, we explicitly consider power and GOP/s/W as primary metrics for comparison, as they directly reflect the relevance of the presented architecture for long-endurance USV missions where autonomy and energy budget are major system-level constraints.

## 6. Discussion

This section discusses the performance of the proposed accelerator in the light of the quantitative results reported in Section 5 and the comparative data summarised in Table 7. The goal is to position our VHDL-based streaming architecture with respect to existing CNN–FPGA designs on FPGA-SoCs, to identify the main architectural trade-offs on a low-cost Zynq-7020, and to assess the relevance of the achieved performance for embedded unmanned surface vehicle (USV) vision workloads. In addition to throughput and latency, Table 7 now reports the energy efficiency (GOP/s/W), which is a key metric for long-endurance USV missions where SWaP constraints and battery autonomy dominate system-level design choices.

Table 7 compares the proposed accelerator with several representative FPGA-based CNN designs covering different device classes, precisions and architectural styles. A first observation is the clear separation between accelerators implemented on high-end devices (Zynq-7045, Kintex-7, UltraScale+) and those targeting the low-cost Zynq-7020. Angel-Eye [27], NullHop [28], the FPGA-SoC CNN inference design in [31] and the low-end FPGA accelerator in [32] all exploit large 7-series FPGAs with abundant LUT, BRAM and DSP resources, combined with 8–16-bit fixed-point or low-bit quantisation and wide processing-element arrays. These architectures report VGG16 frame rates between 4.4 and 11.8 FPS and effective throughputs in the 15.7–425.3 GOP/s range, at the price of very high resource usage (up to 183 k LUTs, 128 k flip-flops and 780 DSPs on Zynq-7045). Overall, the reported performance highlights what is achievable when the device budget is generous and aggressive quantisation is adopted, and they define a design space that is difficult to transpose unchanged to mid-range SoCs.

Several accelerators cluster around the same effective throughput range as the proposed design. On Zynq-7020, fpgaConvNet [26] and mNet2FPGA [30] both use 16-bit fixed-point arithmetic with 220 and 68 DSP slices, respectively; fpgaConvNet reports 48.5 GOP/s for the VGG16 convolutional layers, while the end-to-end VGG16 latencies of mNet2FPGA correspond to 0.27 FPS and about 8.3 GOP/s. NullHop [28] on a Zynq-7100 achieves 0.44 FPS and 17.2 GOP/s at 2.34 W with 16-bit precision and 128 DSPs, and the CNNA architecture of Bjerge et al. [29] on an Ultra96 (ZU3EG) reaches roughly 0.51 FPS and 15.7 GOP/s at 2.63 W with 16-bit fixed point. Under the same assumptions, the proposed accelerator attains 0.29 FPS and 8.9 GOP/s on Zynq-7020. All these designs therefore operate in a similar throughput regime, although they differ significantly in resource and device profiles. Our architecture uses only 36 DSP48E1 slices (about half the DSP budget of mNet2FPGA and far fewer than NullHop or CNNA) while adopting a wider 32-bit fixed-point datapath. The LUT and flip-flop utilisation (45.0 k and 44.3 k) remains compatible with the Zynq-7020, and the estimated PL power of 2.04 W is close to the 2.13 W reported in [30]. These results indicate that in the 8–16 GOP/s range the proposed streaming datapath offers a favourable balance between numerical precision, DSP usage, device size and end-to-end throughput. From an energy perspective, the proposed design reaches about 4.36 GOP/s/W, which is comparable to mNet2FPGA (3.90 GOP/s/W) and within the same order of magnitude as other fixed-point streaming accelerators targeting embedded deployments (e.g., NullHop at 7.35 GOP/s/W and CNNA at 5.97 GOP/s/W when power is reported). On newer UltraScale+ platforms, CNNA [29] illustrates the level of performance that can be reached with an HLS/SystemC-generated engine operating at 172 MHz and using 16-bit fixed-point arithmetic. CNNA processes VGG16 in about 2.0 s at 2.63 W, corresponding to 15.7 GOP/s and 6.0 GOP/s/W, approximately twice the 8.9 GOP/s obtained here on a Zynq-7020 at 100 MHz and 32-bit fixed point, with the benefits of a newer 16 nm MPSoC and a larger effective compute budget. This cross-device scaling suggests that the proposed streaming organisation is sound and that higher throughput can be obtained by migrating the same datapath principles to a device with a larger LUT budget and/or by reducing precision to lower memory traffic and increasing compute density.

The scaling behaviour across networks in Table 5 also supports the validity of the presented architecture. Assuming an effective budget of about 4.45 GMAC/s (8.9 GOP/s), the measured frame rates for VGG16, AlexNet and ResNet-18 closely follow their operation counts, which indicates that the streaming datapath and PS/PL orchestration do not introduce dominant or irregular overheads. The VGG16 latency breakdown further shows that roughly 72% of the time is spent in PL computation (Conv/Pool and FC), whereas the remaining 28% is associated with DMA transfers and PS-side control. This confirms that the architecture is compute-centric and that future gains should primarily come from improving data-movement scheduling and reducing software orchestration overhead (e.g., layer-to-layer overlap, double buffering, and reduced cache-maintenance costs under PYNQ), which would increase both FPS and GOP/s/W under the same power envelope.

In addition to the FPGA-focused comparison of Table 7, it is also useful to place the proposed Zynq-7020 accelerator in the broader landscape of embedded platforms commonly used in maritime vision systems, such as NVIDIA Jetson modules and low-cost single-board computers. This cross-platform perspective is relevant for USV payloads, where system-level choices are driven by SWaP constraints and long-endurance operation. To this end, Table 8 provides a concise cross-platform view. In particular, our own maritime detection pipelines [20,34] contextualise the detector-centric requirements of USV perception and illustrate practical end-to-end performance on embedded GPU platforms, whereas NVIDIA documentation provides representative power configurations for embedded modules (e.g., AGX Xavier power profiles) [23].

A direct one-to-one comparison across heterogeneous embedded platforms is inherently challenging. Reported performance figures can vary substantially with the workload definition (classification backbone vs. end-to-end detector), input resolution, software stack and optimisation level (e.g., TensorRT/CUDA vs. CPU inference), and the adopted power-reporting methodology (module power modes vs. board-level or application-level measurements). For this reason, Table 8 is not presented as a strict FPS leaderboard. Instead, it provides a power-oriented, system-level snapshot of representative embedded hardware commonly used for maritime vision from CPU-only single-board computers (e.g., Raspberry Pi 4B) to embedded GPU modules (Jetson Nano/TX2) and positions our low-cost Zynq-7020 FPGA-SoC prototype within this practical SWaP-driven design space. In this landscape, the proposed Zynq-7020 streaming accelerator targets a complementary operating point characterised by a tightly bounded power budget (about 2 W on-chip in the present prototype), deterministic datapath execution enabled by a fully streaming RTL implementation, and near-sensor processing capability.

Our continued use of VGG-style backbones in this paper was motivated by two factors: (i) controlled and reproducible architectural benchmarking on a resource-constrained SoC, and (ii) direct comparability with a substantial body of FPGA–CNN acceleration literature (Table 7). At the same time, the maritime YOLO references [20,34] included in Table 8 contextualise modern detector-centric requirements in operational USV settings and strengthen the practical relevance of the study. While the present prototype primarily emphasises energy-efficient and predictable execution, improving the end-to-end frame rate remains an important direction; as discussed in the future-work section, this can be pursued through system-level and architectural optimisations such as reduced-precision datapaths, lower DDR/DMA traffic, and improved compute–communication overlap, and by extending the same streaming building blocks toward lightweight detector backbones under the same SWaP envelope.

These characteristics are particularly relevant for USV perception. In typical surface-navigation scenarios, platform speeds are relatively low compared with aerial vehicles, yet onboard energy budgets remain limited and communication with a control station often relies on a narrowband datalink. Under such constraints, a low-power near-sensor inference core can reduce both communication load and system energy by executing the convolutional workload locally and transmitting only compact decisions or metadata. With a PL power of about 2 W and multi-frame-per-second throughput for medium-complexity CNNs such as AlexNet and ResNet-18, the proposed Zynq-7020 accelerator offers a favourable sensing–energy compromise, while maintaining a compact hardware footprint compatible with embedded maritime platforms. In this sense, the architecture presented in this work is not only a competitive CNN accelerator on a small FPGA, but also a practical near-sensor processing building block for energy-aware USV vision systems where energy efficiency and predictable latency are key determinants of mission autonomy.

### Limitations and Future Work

The proposed ARM–FPGA streaming co-design was intentionally evaluated in a controlled and reproducible setting to quantify the impact of computation mapping, memory traffic and PS/PL orchestration on a low-cost Zynq-7020 platform. This positioning defined clear boundaries that help interpret the reported results and guide the next development steps.

First, the experimental validation was conducted on standard classification backbones (VGG16, AlexNet and ResNet-18) trained on SeaShips-derived ship crops, which provided a controlled benchmark for characterising the proposed datapath and quantifying system-level overheads, while enabling fair comparison with prior CNN–FPGA studies; however, operational USV perception typically involves continuous video streams and additional stages such as detection, tracking and temporal filtering, and the end-to-end throughput remains influenced by system-level interactions between PL compute and data movement coordinated by the PS, especially for large networks and multi-stage pipelines. Future work will therefore focus on extending the near-sensor core toward an end-to-end USV perception pipeline under the same SWaP envelope, and on optimising buffering, transfer scheduling and compute–communication overlap to increase FPS while preserving high energy efficiency on low-cost FPGA platforms such as the Zynq-7020.

In addition, future optimisation will investigate lower-precision datapaths (e.g., 16-bit and 8-bit quantisation), with a dedicated study to quantify the accuracy degradation versus the expected gains in resource usage and data-movement efficiency. In particular, reducing tensor word width is expected to directly affect DDR/DMA traffic, effective bandwidth utilisation on the HP0 interface, and compute–transfer overlap; therefore, we will report network-level accuracy metrics together with end-to-end latency and bandwidth measurements under multiple precisions to identify the best precision point for the Zynq-7020 deployment.

## 7. Conclusions

This paper investigated a VHDL-based convolutional neural network accelerator implemented on a low-cost Xilinx Zynq-7020 SoC (ZedBoard) and positioned it as a near-sensor processing core for ship recognition in unmanned surface vehicles. The presented architecture combines a fully streaming dataflow with line-buffer-based convolutions, max-pooling and fully connected layers interconnected via AXI-Stream, while the ARM processing system running PYNQ is confined to configuration, orchestration and AXI DMA data transfers, resulting in a clean and reproducible hardware–software partitioning. Our post place-and-route results confirm that the accelerator fits comfortably within the limited resources of the Zynq-7020, using 45,035 LUTs, 44,306 flip-flops, 58 BRAM36 blocks and only 36 DSP48E1 slices, and meeting timing at 100 MHz with an estimated on-chip power of about 2.04 W. When evaluated on VGG16, AlexNet and ResNet-18 trained on the SeaShips dataset, the design delivered an effective throughput of approximately 8.9 GOP/s and end-to-end frame rates of 0.29 FPS, 6.15 FPS and 4.94 FPS, respectively, thereby providing multi-frame-per-second inference for medium-complexity CNNs on a modest SoC. A comparison with representative CNN–FPGA accelerators on Xilinx 7-series and UltraScale+ devices indicated that the proposed design offers a favourable balance between numerical precision, DSP usage, device size and throughput, and demonstrates that a compact Zynq-7020 platform can deliver practical multi-frame-per-second CNN inference for embedded maritime perception under strict size, weight and power constraints.

## Figures and Tables

**Table 1:** Class distribution of ship instances derived from the SeaShips dataset.

Class	Number of Crops	Share [%]
Ore carrier	2200	23.9
Bulk cargo carrier	1953	21.2
Container ship	1505	16.3
General cargo ship	901	9.8
Fishing boat	2190	23.7
Passenger ship	474	5.1
Total	9223	100

**Table 2:** Comparison between a sequential MAC-based convolution engine and the proposed parallel convolution core for a 3×3 kernel.

Metric	Sequential MAC-Based Core	Proposed Parallel Core
Multipliers per 3×3 kernel	1	9
Adders for kernel sum	1 (re-used sequentially)	8 (plus bias addition)
Cycles per output pixel (3×3)	≈9	≈1
Relative throughput	baseline	≈9× higher
Line-buffer organisation	*K* line buffers of length $W_{\mathrm{in}}$; kernel coefficients processed sequentially by a single MAC unit	*K* line buffers of length $W_{\mathrm{in}}$; window re-used while producing one output per cycle (after priming)

**Table 3:** DDR memory regions allocated by the PYNQ runtime for CNN inference.

Region	Content (32-bit Q15.16 Words, Contiguous in DDR)
IMG_BASE	Input image/input feature map buffer (ping)
FM_BASE0	Intermediate feature-map buffer (pong)
FM_BASE1	Intermediate feature-map buffer (ping)
W_BASE(l)	Weights of layer *l* flattened as in (7)
B_BASE(l)	Bias vector of layer *l*
FCW_BASE	Fully connected weights (if enabled), contiguous per output neuron
FCB_BASE	Fully connected biases
RES_BASE	Final classification scores/output vector

**Table 4:** Post place-and-route resource utilisation of the proposed CNN accelerator on the Zynq-7020 (XC7Z020).

Resource	Used	Available	Utilisation [%]
LUT	45,035	53,200	84.65
LUTRAM	2523	17,400	14.50
Flip-Flops	44,306	106,400	41.64
BRAM36	58	140	41.43
DSP48E1	36	220	16.36
Clock frequency	100 MHz (PL)		
On-chip power (estimate)	2.04 W		

**Table 5:** End-to-end latency and throughput of the proposed accelerator.

Model	GMAC/Image	Latency [ms]	FPS
VGG16	15.47	3476	0.29
AlexNet	0.724	163	6.15
ResNet-18	0.90	202	4.94

**Table 6:** Latency breakdown for a VGG16 inference.

Component	Latency [ms]	Share [%]
Conv + Pool (13 blocks, PL)	1534	44.1
Fully connected	972	28.0
DMA data transfers (AXI HP0, 64-bit)	488	14.0
PS control (DMA setup, AXI-Lite, Python)	374	10.8
Image I/O and result write-back	108	3.1
Total	3476	100

**Table 7:** Comparison of FPGA-based accelerators.

Metric	fpgaConvNet	Angel-Eye	NullHop	mNet2FPGA	Spagnolo et al.	Bjerge et al. (CNNA)	Shen et al.	This Work
	[26]	[27]	[28]	[30]	[31]	[29]	[32]	–
Year	2019	2018	2019	2020	2020	2021	2025	2025
FPGA device	Zynq-7020	Zynq-7045	Zynq-7100	Zynq-7020	Zynq-7045 (ZC706)	Zynq UltraScale+ ZU3EG (Ultra96)	Zynq-7045 (ZC706)	Zynq-7020 (ZedBoard)
LUTs [k]	n/a	183	229	36	30.2	n/a	n/a	45.0
Flip-flops [k]	n/a	128	107	43	47.9	n/a	n/a	44.3
BRAM [Mb]	n/a	2.14	1.78	1.54	n/a	n/a	n/a	2.04
DSPs	220	780	128	68	880	360	n/a	36
Power [W]	n/a	9.63	2.34	2.13	n/a	2.63	n/a	2.04
Frequency [MHz]	125	150	60	50/100	167	172	200	100
Precision	16-bit fixed	8-bit fixed	16-bit fixed	16-bit fixed	8/16-bit fixed	16-bit fixed	Low-bit quantised	32-bit fixed
FPS	n/a	n/a	0.44	0.27	11.8	0.51	4.45	0.29
GOP/s	48.53	137.0	17.2	8.3	425.3	15.7	136.6	8.9
Energy efficiency [GOP/s/W]	n/a	14.2	7.35	3.90	n/a	5.97	n/a	4.36

**Table 8:** Cross-platform comparison across embedded platforms.

Platform	Accel.	Model/Workload	FPS	Power [W]
Raspberry Pi 4B [65,66]	CPU	YOLOv8	3.1	7.6
Jetson Nano [67]	GPU	YOLOv8	14.5	5/10
Jetson TX2 [20]	GPU	YOLOv8	17.99	7.5
This work (Zynq-7020/ZedBoard)	FPGA-SoC	VGG16	0.29	2.04

## Data Availability

Data are contained within the article.

## References

[1] Manley J.E. (2008). Unmanned Surface Vehicles, 15 Years of Development. Proceedings of the MTS/IEEE OCEANS.

[2] Caccia M., Bibuli M., Bono R., Bruzzone G. (2008). Basic Navigation, Guidance and Control of an Unmanned Surface Vehicle. Auton. Robot..

[3] Bae I., Hong J. (2023). Survey on the Developments of Unmanned Marine Vehicles: Intelligence and Cooperation. Sensors.

[4] Sotelo–Torres F., Alvarez L.V., Roberts R.C. (2023). An Unmanned Surface Vehicle (USV): Development of an Autonomous Boat with a Sensor Integration System for Bathymetric Surveys. Sensors.

[5] Prasad D.K., Rajan D., Rachmawati L., Rajabally E., Quek C. (2016). Video Processing from Electro-Optical Sensors for Object Detection: A Survey. IEEE Trans. Intell. Transp. Syst..

[6] LeCun Y., Bengio Y., Hinton G. (2015). Deep Learning. Nature.

[7] Krizhevsky A., Sutskever I., Hinton G.E. (2012). ImageNet Classification with Deep Convolutional Neural Networks. NeurIPS 2012.

[8] Simonyan K., Zisserman A. (2015). Very Deep Convolutional Networks for Large-Scale Image Recognition. ICLR 2015.

[9] Redmon J., Divvala S., Girshick R., Farhadi A. (2016). You Only Look Once: Unified, Real-Time Object Detection. 2016 IEEE Conference on Computer Vision and Pattern Recognition (CVPR).

[10] Liu W., Anguelov D., Erhan D., Szegedy C., Reed S., Fu C.Y., Berg A.C. (2016). SSD: Single Shot MultiBox Detector. ECCV 2016.

[11] Ren S., He K., Girshick R., Sun J. (2015). Faster R-CNN: Towards Real-Time Object Detection with Region Proposal Networks. NeurIPS 2015.

[12] He K., Zhang X., Ren S., Sun J. (2016). Deep Residual Learning for Image Recognition. 2016 IEEE Conference on Computer Vision and Pattern Recognition (CVPR).

[13] Shao Z., Wu W., Wang Z., Du W., Li C. (2018). SeaShips: A Large-Scale Precisely Annotated Dataset for Ship Detection. IEEE Trans. Multimed..

[14] Varga L.A., Kiefer B., Meßmer M., Zell A. (2022). SeaDronesSee: A Maritime Benchmark for Detecting Humans in Open Water. 2022 IEEE/CVF Winter Conference on Applications of Computer Vision (WACV).

[15] Iancu B., Soloviev V., Zelioli L., Lilius J. (2021). Aboships—A Near-Shore and Offshore Maritime Vessel Detection Dataset with Precise Annotations. Remote Sens..

[16] Zhang S., Wu R., Xu K., Wang J., Sun X. (2019). R-CNN-Based Ship Detection from High-Resolution Remote Sensing Imagery. Remote Sens..

[17] Zhao H., Liu X., Liu L., Lin W., Li S., Ma C. (2023). YOLOv7-Sea: Object Detection of Maritime UAV Images Based on Improved YOLOv7. WACV Workshops.

[18] Li L., Chen Z., Han W., Meng X., Wang P. (2024). Spotlight on Small-Scale Ship Detection: Empowering YOLO with Advanced Techniques. Proceedings of the Asian Conference on Computer Vision (ACCV).

[19] Zhao Q., Xu J., Sun Y., Wang Y., Wu Z. (2024). E2YOLOX-VFL: Multi-Scale Ship Detection in Optical Remote Sensing Images. Remote Sens..

[20] Haijoub A., Hatim A., Guerrero-Gonzalez A., Arioua M., Chougdali K. (2024). Enhanced YOLOv8 Ship Detection Empower Unmanned Surface Vehicles for Advanced Maritime Surveillance. J. Imaging.

[21] Sze V., Chen Y.H., Yang T.J., Emer J.S. (2017). Efficient Processing of Deep Neural Networks: A Tutorial and Survey. Proc. IEEE.

[22] Hennessy J.L., Patterson D.A. (2017). Computer Architecture: A Quantitative Approach.

[23] NVIDIA (2020). NVIDIA Jetson AGX Xavier Series Product Design Guide.

[24] Jouppi N.P., Young C., Patil N., Patterson D., Agrawal G., Bajwa R., Bates S., Bhatia S., Boden N., Borchers A. (2017). Datacenter Performance Analysis of a Tensor Processing Unit.

[25] Shawahna A., Sait S.M., El–Maleh A. (2019). FPGA-Based Accelerators of Deep Learning Networks: A Review. IEEE Access.

[26] Venieris S.I., Bouganis C.-S. (2019). fpgaConvNet: Mapping Regular and Irregular Convolutional Neural Networks on FPGAs. IEEE Trans. Neural Netw. Learn. Syst..

[27] Guo K., Zeng S., Yu J., Wang Y., Yang H. (2018). Angel-Eye: A Complete Design Flow for Mapping CNN onto Embedded FPGA. IEEE Trans. Comput.-Aided Des. Integr. Circuits Syst..

[28] Aimar A., Lungu I., Rios-Navarro A., Camuñas-Mesa L.A., Benatti S., Rossi D., Digioia M., Ceolini E., Delbruck T., Benini L. (2019). NullHop: A Flexible Convolutional Neural Network Accelerator Based on Sparse Representations for Embedded Systems. Front. Neurosci..

[29] Bjerge K., Schougaard J.H., Larsen D.E. (2021). A Scalable and Efficient Convolutional Neural Network Accelerator Using HLS for a System-on-Chip Design. Microprocess. Microsyst..

[30] Sledevič T., Navickas R., Kriščiūnas T., Juršēnas A. (2020). mNet2FPGA: A Design Flow for Mapping a Fixed-Point CNN to Zynq SoC FPGA. Electronics.

[31] Spagnolo F., Tota S., Masera G., Martina M. (2020). Energy-Efficient Architecture for CNNs Inference on Heterogeneous FPGA-SoC Devices. J. Low Power Electron. Appl..

[32] Shen J., Yuan X., Hu Z., Li K., Chen S., Li M., Luo H., Wang H., Wang K., Duan J. (2025). Efficient CNN Accelerator Based on Low-End FPGA with Optimized Depthwise Separable Convolutions and Squeeze-and-Excite Modules. AI.

[33] Valdenegro-Toro M. (2019). MODS: A Maritime Object Detection Dataset for Optical Remote Sensing Images. arXiv.

[34] Haijoub A., Hatim A., Arioua M., Hammia S., Eloualkadi A., Guerrero-González A., Essaaidi M., Morales-Bueno R. (2023). Fast YOLOv7-Based CNN for Video Streaming Sea Ship Recognition and Sea Surveillance. Modern Artificial Intelligence and Data Science.

[35] Gao L., Luo Z., Wang L. (2025). Convolutional Neural Network Acceleration Techniques Based on FPGA Platforms: Principles, Methods, and Challenges. Information.

[36] Alqahtani D.K., Cheema A., Toosi A.N. (2024). Benchmarking Deep Learning Models for Object Detection on Edge Computing Devices. arXiv.

[37] Venieris S.I., Bouganis C.S. (2018). Toolflows for Mapping Convolutional Neural Networks on FPGAs: A Survey and Future Directions. ACM Comput. Surv..

[38] Ghaffari A., Niar S., Abid M. (2021). FPGA-Based Acceleration of Deep Neural Networks: A Review. Electronics.

[39] Wu R., Guo X., Du J., Li J. (2021). Accelerating Neural Network Inference on FPGA-Based Platforms—A Survey. Electronics.

[40] Huang J., Liu X., Guo T., Zhao Z. (2023). A High Performance FPGA-Based Accelerator for CNN-Based Depthwise Separable Convolution. Electronics.

[41] Wan Y., Chen J., Yang X., Zhang H., Huang C., Xie X. (2025). DSA-CNN: An FPGA-Integrated Deformable Systolic Array for Convolutional Neural Network Acceleration. Appl. Intell..

[42] Zhang Z., Mahmud M.A.P., Kouzani A.Z. (2022). Resource-Constrained FPGA Implementation of YOLOv2. Neural Comput. Appl..

[43] Tesema S.N., Bourennane E.-B. (2022). Resource- and Power-Efficient High-Performance Object Detection Inference Acceleration Using FPGA. Electronics.

[44] Fang N., Li L., Zhou X., Zhang W., Chen F. (2025). An FPGA-Based Hybrid Overlapping Acceleration Architecture for Small-Target Remote Sensing Detection. Remote Sens..

[45] Li B., Yang L., Song J., Chen H., Wu S., Li T., Liang Y., Zhang J. (2025). Optimized FPGA Architecture for CNN-Driven Subsurface Geotechnical Defect Detection. Electronics.

[46] Zheng X., Wei H., Peng L., Zhao X., Hu H., Gao M., Yan W. (2025). FPGA-Based Low-Power High-Performance CNN Accelerator Integrating DIST for Rice Leaf Disease Classification. Electronics.

[47] Gundrapally A., Shah Y.A., Alnatsheh N., Choi K.K. (2024). A High-Performance and Ultra-Low-Power Accelerator Design for Advanced Deep Learning Algorithms on an FPGA. Electronics.

[48] Jacob B., Kligys S., Chen B., Zhu M., Tang M., Howard A., Adam H., Kalenichenko D. (2018). Quantization and Training of Neural Networks for Efficient Integer-Arithmetic-Only Inference. IEEE Conference on Computer Vision and Pattern Recognition (CVPR).

[49] Rastegari M., Ordonez V., Redmon J., Farhadi A. (2016). XNOR-Net: ImageNet Classification Using Binary Convolutional Neural Networks.

[50] Zhao R., Li W., You H., Guo Y., He B., Zhang W. (2019). Accelerating Binarized Convolutional Neural Networks on FPGAs.

[51] Umuroglu Y., Fraser N.J., Gambardella G., Blott M., Leong P., Jahre M., Vissers K. (2017). FINN: A Framework for Fast, Scalable Binarized Neural Network Inference.

[52] Chen Y.-H., Krishna T., Emer J.S., Sze V. (2016). Eyeriss: An Energy-Efficient Reconfigurable Accelerator for Deep Convolutional Neural Networks. IEEE J. Solid-State Circuits.

[53] Chen Y.-H., Yang T.-J., Emer J.S., Sze V. (2018). Eyeriss v2: A Flexible Accelerator for Emerging Deep Neural Networks on Mobile Devices. IEEE J. Emerg. Sel. Top. Circuits Syst..

[54] Du L., Li Q., Xu M., Zhang Z., Wang J. (2020). A Reconfigurable CNN Accelerator with Multi-Row Line Buffers. Electronics.

[55] Zhang C., Li P., Sun G., Guan Y., Xiao B., Cong J. (2017). Optimizing FPGA-Based Accelerator Design for Deep CNNs.

[56] Ma Y., Cao Y., Vrudhula S., Seo J.-S. (2017). Optimizing Loop Operation and Dataflow in FPGA Acceleration of Deep CNNs.

[57] Kang H.-J., Yang B.-D. (2023). AoCStream: All-on-Chip CNN Accelerator with Stream-Based Line-Buffer Architecture and Accelerator-Aware Pruning. Sensors.

[58] MacLellan A., Crockett L.H., Stewart R.W. (2023). Streaming Convolutional Neural Network FPGA Architecture for RFSoC Data Converters. 2023 IEEE International New Circuits and Systems Conference (NEWCAS).

[59] Gschwend D. (2020). ZynqNet: An FPGA-Accelerated Embedded Convolutional Neural Network. arXiv.

[60] Park S., Kim S., Lee D., Choi J., Kim H. (2019). CloudSatNet: FPGA-Based Satellite Image Classification Accelerator. IEEE Access.

[61] Pitonak R., Mucha J., Dobis L., Javorka M., Marusin M. (2022). CloudSatNet-1: FPGA-Based Hardware-Accelerated Quantized CNN for Satellite On-Board Cloud Coverage Classification. Remote Sens..

[62] Neelam S., Amalin P.A. (2025). VCONV: A Convolutional Neural Network Accelerator for FPGAs. Electronics.

[63] Haijoub A., Hatim A., Arioua M., Hammia S., Eloualkadi A., Guerrero-González A. (2023). Implementing Convolutional Neural Networks on FPGA: A Survey and Research. ITM Web Conf..

[64] Johnson C., Hauck S., Hsu S.-C., Khan W., Bavier M., Kondratyuk O., Nguyen T., Ayala-Cerna S., Short A., Silva J. (2023). Quantifying the Efficiency of High-Level Synthesis for Machine Learning Inference. Proceedings of the IEEE/ACM International Conference on Computer-Aided Design (ICCAD).

[65] Pidramble Power Consumption Benchmarks (Raspberry Pi 4 Idle/Load Measurements). https://www.pidramble.com/wiki/benchmarks/power-consumption.html.

[66] TomsHardware (2022). Raspberry Pi 4 Review (Power Under Load).

[67] NVIDIA (2019). Jetson Nano Developer Kit User Guide.

